# Translation, Cultural Adaptation, and Mixed-Methods User Feedback of the SelfBack App to the Arabic Language for Patients With Low Back Pain: Pilot Mixed-Methods Study

**DOI:** 10.2196/59777

**Published:** 2025-07-25

**Authors:** Mai Aldera, Hana Alsobayel, Jonathan Hill, Christian Lodberg Jensen

**Affiliations:** 1 Department of Rehabilitation Science, College of Applied Medical Science, King Saud University, P. O. Box 800, Riyadh, Saudi Arabia, +966 501122660; 2 School of Medicine, Keele University, Keele, United Kingdom; 3 SelfBack, Odense, Denmark

**Keywords:** low back pain, self-management, treatment, digital intervention, quality of life, language, smartphone, mobile app, Arabic-speaking, Arabic translation, cultural adaptation, cohort, mixed methods, user feedback, mobile health, usability, interview, Saudi Arabia

## Abstract

**Background:**

Low back pain (LBP) is a pervasive global health concern, significantly impacting the quality of life and burdening health care systems. Effective self-management strategies are essential for mitigating the effects of LBP and empowering individuals in their recovery. Digital health interventions, particularly smartphone apps, offer a promising avenue for delivering accessible and personalized self-management support, potentially improving patient adherence to treatment plans. The SelfBack app exemplifies such digital innovations, demonstrating clinical effectiveness in LBP management and prioritizing personalized user experiences.

**Objective:**

This study describes the comprehensive process of translating and culturally adapting the SelfBack app from English to Arabic for the Saudi context. It further evaluates the perceived usability of the adapted Arabic SelfBack app within a cohort of Saudi individuals experiencing LBP, aiming to ensure accessibility and cultural appropriateness for Arabic-speaking users.

**Methods:**

A rigorous, 5-stage process was implemented. Stage 1, exchange, focused on adapting the app’s core content. English self-assessment scales, used for tailored treatment plans, were replaced with validated, culturally appropriate Arabic versions. Stage 2, translation and cultural adaptation, which translated the content of the patient self-management plan and adapted it for cultural relevance. Stage 3, audio conversion, addressed educational resources. English audio content was professionally translated and rerecorded in Arabic. Stage 4, laboratory usability testing, integrated the Arabic content, verifying interface functionality and right-to-left script compatibility. Stage 5*,* field usability testing, evaluated the app with 11 Saudi participants experiencing nonspecific LBP using the Arabic System Usability Scale (A-SUS) and semistructured interviews.

**Results:**

The translation and adaptation processes are detailed, highlighting the work of an expert panel of linguists, health care professionals, and cultural consultants. The panel identified minimal discrepancies and no significant misunderstandings, demonstrating the accuracy and cultural appropriateness of the adaptation. The mean System Usability Scale (SUS) score was 70%, indicating good usability. Interviews corroborated these results, with participants generally reporting the app as clear, intuitive, and easy to use. However, feedback highlighted areas for improvement, including the perceived number of mandatory questions, a perceived lack of interactivity, repetitive content, and unmet expectations regarding certain functionalities.

**Conclusions:**

The Arabic SelfBack app has been successfully developed, demonstrating high user satisfaction, ease of use, and interface efficiency within the target cultural context. The identified criticisms provide actionable insights for future updates. These results suggest that the app is ready for clinical research with Arabic-speaking participants, potentially improving LBP management in Saudi Arabia. Further research will be essential to confirm these initial findings and establish the app’s long-term effectiveness.

## Introduction

Low back pain (LBP) is a significant global health concern, adversely impacting a significant portion of the population throughout their lives [1]. It ranks among the leading causes of disability in adults and generates a substantial public health burden due to its impact on affected individuals’ well-being and quality of life [2]. The prevalence of LBP is particularly concerning, with estimates suggesting that up to 80% of adults will experience LBP at some point in their lives [1]. This underscores the need for effective and accessible interventions.

Clinical guidelines advocate that patients with LBP be provided with supported self-management strategies to empower them to manage their pain. These strategies include exercise, education, pain relief, lifestyle advice around sleep, diet, and stress, as well as advice regarding work and maintaining normal activities [3]. However, people with LBP often find it hard to implement these strategies due to lack of knowledge, fears about doing the wrong thing, lack of confidence, competing routines, feeling overwhelmed by information, limited access to physical activity resources, lack of trust in what to do, and lack of feedback about the benefits of adhering to their treatment regimens [4]. These barriers highlight the gap between recommended care and actual practice.

Digital interventions, including smartphone apps, have emerged as promising solutions to overcome these challenges [5]. They offer evidence-based and cost-effective methods for delivering self-management solutions for people with LBP [4]. Digital health interventions can increase access to care, particularly in remote areas or for individuals with mobility limitations. They also offer the potential for personalized interventions tailored to individual needs and preferences [6].

While a systematic review found that numerous LBP apps are available, their overall quality was often poor, characterized by a lack of engaging features, unattractive interfaces, and unreliable information [7]. This highlights the importance of rigorous evaluation and quality assurance in the development and deployment of LBP apps. The recent National Institute for Health and Care Excellence (NICE) Early Value Assessment of digital self-management apps for LBP [8] identified 10 apps, 5 of which were recommended for use in the National Health Service (NHS), including SelfBack, and 5 that can only be used in research. The NICE evaluation underscores the growing potential of digital tools in chronic pain management but emphasizes the need for rigorous assessment before mainstream integration. This evaluation process provides a valuable framework for assessing the clinical and cost-effectiveness of digital health interventions.

Various smartphone apps, including Spine Zone [9], Pain Coach [10], and getUBetter [11], address LBP self-management. The SelfBack app stands out for its unique evidence-based approach and dedication to personalized user experiences. Its efficacy has been shown in a large randomized controlled trial [4], where participants using SelfBack demonstrated significant reductions in pain and disability compared to a control group after 3 months. This robust evidence base distinguishes SelfBack from many other LBP apps. Furthermore, its personalized approach, based on individual assessments and feedback, may contribute to improved engagement and adherence.

At present, there are no digital tools available in the Arabic language for supporting self-management for people with LBP. Other self-management apps for different health conditions, such as the Sehhaty app (the Saudi governmental health platform) [12] and the Sehhaty Wa Daghty app, have demonstrated effectiveness in the Saudi cultural context [13]. These successful examples of digital health interventions in Saudi Arabia highlight the potential for culturally adapted LBP apps. Therefore, the development of an app supporting self-management for Arabic-speaking people with LBP is important. However, translating software app content involves much more than just basic word-for-word translation. It requires a nuanced approach that considers cultural context, user experience, and technical limitations. The objectives of this study were, therefore, to translate and culturally adapt the English language version of the SelfBack app to the Arabic language and evaluate user satisfaction.

## Methods

### Design

A prospective study was conducted to assess user satisfaction with the SelfBack app after its translation and cultural adaptation from English to Arabic.

### The SelfBack App

**Figure 1.**
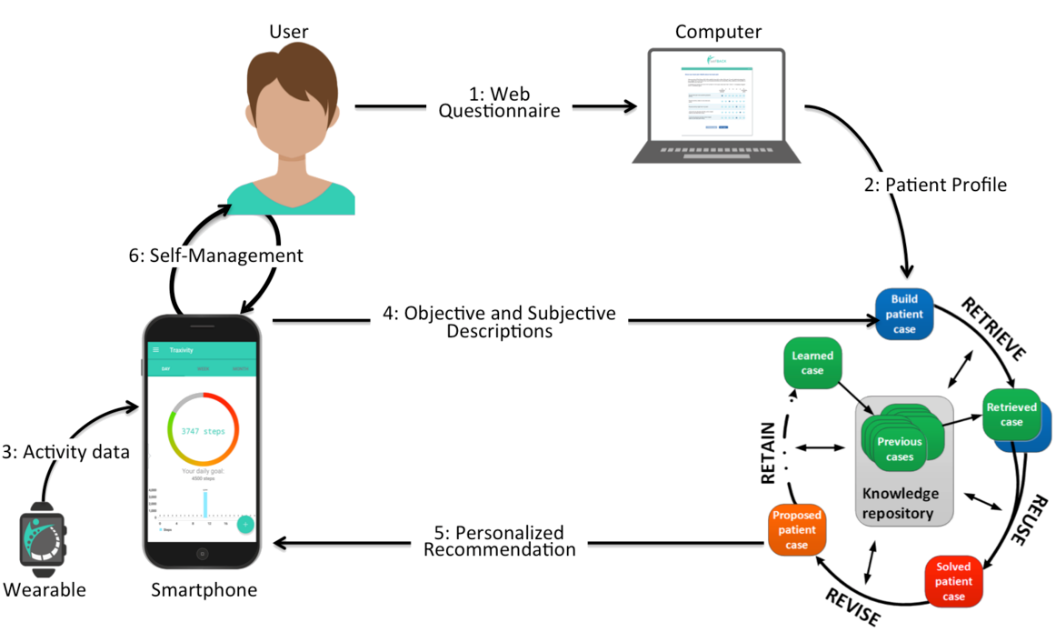
SelfBack app workflow. (adapted from SelfBack website [14].

The SelfBack app is a smartphone app that uses artificial intelligence to create personalized self-management plans for people with nonspecific LBP based on individual person data within a case-based reasoning system, as shown in Figure 1 [4]. It is designed for self-management and uses an evidence-based decision support system. The app works by first asking the person to fill out a range of scales and questionnaires about their LBP condition. For example, it asks about their pain severity, functional ability, fear avoidance behaviors, workability, perceived barriers to self-management, pain self-efficacy, sleep, perceived stress, and mood. This information is then used to create a personalized treatment plan, which consists of a combination of physical exercises and educational materials.

The treatment plan is updated weekly based on physical activity monitoring and self-reported adherence to the self-management plan. The app uses the person’s smartphone to track their physical activity levels or can link to a wearable step counter if the person has one. This information is used to ensure that the treatment plan is challenging, but not too difficult, and to identify any areas where the person may need additional support.

To use the SelfBack app, a person first needs to download the app from the App Store or Google Play. For the purpose of this research, patients were initially screened by their treating clinician as appropriate, before being signposted to the app and provided with a token to enable the app to be downloaded for free. Once the app was installed on their phone, they created an account and filled out a range of questions about their condition to initiate their individual treatment plan. The treatment plan remains dynamic and depends on the person’s progress, which is adjusted weekly as needed.

### Translation and Cultural Adaptation

The translation and cultural adaptation of the SelfBack app contents from the English language to the Arabic language required a multistep design approach to ensure it was of high quality [15]. The method comprised 5 phases:

#### Exchange Phase

This phase involved exchanging the SelfBack self-assessment scales and questionnaires used at the baseline and weekly question and answer sessions from the English language version to translated and validated Arabic versions. The investigators (HS and MA) identified ready-to-use Arabic-translated scales and, if necessary, obtained permission for their use.

#### Translation and Cultural Adaptation Phase

This phase focused on the text content of the SelfBack patient self-management plan, translating and culturally adapting it into the Arabic language. This was based on the principles described by Wild et al [16] using a multistep approach: preparation, forward translation to the Arabic language, cultural adaptation, quality assurance, comparison of the 2 versions by an expert panel, proofreading, and adding the final Arabic version to the SelfBack app. To ensure the quality of translation, various steps involving translation were undertaken by native Arabic and English speakers at an authorized language translation service center. The cultural adaptation step focused on replacing common English sayings with Arabic sayings (eg, the statement “trouble sleeping” was replaced by “difficulty sleeping”); This was done by expert bilingual physiotherapists (HA and MA). The quality assurance step was carried out by the experts (HS and MA). Comparison of the 2 versions (English and Arabic) was undertaken by an expert panel comprising 2 investigators (HS and MA) and a consultant psychologist who compared the original English version with the translated Arabic version to assess linguistic equivalency and correct spelling, and that the 2 versions were conceptually equivalent. Final proofreading was conducted by an investigator (HS). Adding the final Arabic version to the SelfBack app was undertaken by the software developers of the SelfBack app.

#### Audio Conversion Phase

This phase involved converting the English language audio educational materials to the Arabic language, using the principles described by Wild et al [16], and requiring multiple steps as follows: preparation, forward translation to the Arabic language, cultural adaptation, quality assurance, comparison of the 2 versions by an expert panel, audio recording, proof listening, and adding the final Arabic version to the SelfBack app. These steps were, mostly, undertaken as described for the translation and cultural adaptation phase. However, in this phase, the expert panel selected 1 person out of 3 candidates to record the audio recordings based on their ability to speak clearly, understandably, and in a professional tone.

#### Laboratory Usability Test Phase

This phase addressed the software interface text of all features by allocating the Arabic materials of the SelfBack app to the appropriate place in the app. This phase involved carefully considering the differences between the English and Arabic languages, in particular in terms of the direction of text writing being right to left in Arabic and that it is not possible to divide Arabic words when starting new lines. Thus, in this phase, the main focus of the investigators (HS and MD) was to address the software interface text of all features by allocating the Arabic materials to the appropriate place in the SelfBack app.

#### Clinical Usability Testing Phase

This phase involved assessing the usability of the Arabic SelfBack app for both the Android and iOS operating systems using the validated and translated Arabic version of the System Usability Scale (A-SUS) [17]. The SUS is a questionnaire used to evaluate the usability of a wide variety of new software systems [18]. It comprises 10 questions which are answered using a 5-point Likert scale ranging from “I strongly disagree” to “I strongly agree.” The SUS generates a score (range 0-100) and offers information about overall customer satisfaction and usability [19]. In addition, to assess user experience with the app, one-to-one phone interviews were undertaken using one open-ended question, “What feedback do you have after using the Arabic version of the SelfBack software app?” List 3 important features.”

This study was conducted as part of a pilot randomized controlled trial aimed at translating, culturally adapting, and evaluating the usability of the SelfBack app for Arabic-speaking users in Saudi Arabia (IRB E-22‐7106). Participants for this phase of the study were randomly recruited from the Primary Care Clinic at King Khalid University Hospital in Riyadh. The participants (N=11) represent a random sample of 50% of the control group who used the app and adhered to it for more than 3 months. Inclusion criteria were adult patients (≥18 y) diagnosed with LBP who had used the SelfBack app for more than 3 months. Exclusion criteria encompassed previous back surgery, specific spine pathologies (eg, spinal stenosis and herniated disc), pregnancy-related back pain, and any other clinical contraindication to self-management via the SelfBack app as determined by the treating clinician.

### Data Analysis

The SUS questionnaire data (10 items) were analyzed using IBM SPSS Statistics for Windows (version 25.0; IBM Corp). The SUS yields a global score reflecting perceived usability, with higher scores indicating better usability and lower scores suggesting potential usability problems. A benchmark of 70% is often used to differentiate acceptable usability from areas needing improvement, while scores below 50% are typically indicative of low usability and necessitate further investigation [19].

The SUS comprises 10 items, 5 of which are positively worded (Q1, Q3, Q5, Q7, and Q9) and 5 negatively worded (Q2, Q4, Q6, Q8, and Q10). Scoring involves a 2-step process. First, responses to each item are converted to numerical values: strongly disagree=1, disagree=2, neutral=3, agree=4, and strongly agree=5. Second, intermediate scores are calculated for the odd- and even-numbered items. For the odd-numbered items, the sum of the item scores is subtracted from 5 (*x*=Σ(Q1, Q3, Q5, Q7, Q9) - 5). For the even-numbered items, the sum of the item scores is subtracted from 25 (*y*=25 - Σ(Q2, Q4, Q6, Q8, Q10)). The final SUS score is then calculated using the following formula: SUS score = (*x+y*) * 2.5. This calculation method reflects the 100-point scale of the SUS, where each item contributes a maximum of 10 points [19].

Data from the open question regarding user experience were analyzed using qualitative content analysis by counting the frequency of the created codes in the transcribed text from the phone interviews. A thematic analysis of interview data was conducted manually, and the participants’ answers were translated from Arabic to English by 2 experts (HS and MD).

### Ethical Considerations

This study involving the translation and cultural adaptation of the SelfBack app, along with usability testing, was reviewed and approved by the research committees of the College of Medicine, King Saud University with the approval number E-22‐7106, and the procedures followed were in accordance with the ethical standards of the responsible committee in human experimentation and with the WMA Declaration of Helsinki. While the usability testing involved human participants, the study focused on evaluating the app’s interface and functionality, not on collecting sensitive personal data.

All participants in the usability testing provided informed consent after receiving a detailed explanation of the study’s purpose, procedures, and rights. The Arabic language consent form explained that the data collected would be used solely for evaluating the app’s usability and would not be used for any other purpose. Participants were also informed of their right to withdraw from the study at any time without penalty.

To ensure privacy and confidentiality, all data collected during the usability testing were anonymized. Participant names and other identifying information were removed from all data files. The interview transcripts were coded, and any potentially identifying information was redacted. The anonymized data will be stored securely and will only be accessible to the research team.

Participants in the usability testing did not receive any compensation for their time and contribution to the study. No images of individual participants or users are included in this paper. Therefore, no consent for image use was required.

## Results

### Translation and Cultural Adaptation Phase

The expert panel participants during the translation and cultural adaptation phases did not report any misunderstandings and only a minor few discrepancies with the translated and culturally adapted instructions and educational materials.

### Clinical Usability Testing

Eleven people with nonspecific LBP participated in the field usability testing. As shown in Table 1, there were 10 (91%) females with ages ranging from 31‐ to 58 years.

**Table 1.** Demographic data of the participants (N=11) who completed 3 months using the SelfBack app, included in the field usability test.

Participant	Sex	Age (y)	MSK-HQ^a^	StartBack tool^b^	Physical activity level (d/wk)
1	Female	37	29	High	4
2	Female	40	46	Low	2
3	Female	36	41	Low	0
4	Female	58	43	Medium	2
5	Female	38	35	High	0
6	Female	52	32	Medium	5
7	Female	38	30	High	2
8	Female	38	38	Medium	0
9	Female	31	44	Low	5
10	Female	38	35	Medium	2
11	Male	31	30	Low	2

a MSK-HQ: Musculoskeletal Health Questionnaire.

b Keel StartBack Tool (the grade level indicates high, medium, and low levels of risk of chronicity and disability).

#### Arabic System Usability Scale

The mean (SD) A-SUS score was 70% (SD 5), reflecting a “good” level of usability [18]. This has been driven by 2 steps: first, convert the scale into numerical values for each of the 10 questions, as follows: strongly disagree: 1 point, disagree: 2 points, neutral: 3 points, agree: 4 points, and strongly agree: 5 points. Then, calculate the scores as the following formula: SUS score=(x+y) * 2.5, where (x) is the sum of the item scores and is subtracted from 5 for all odd-numbered questions, and (y) is the sum of the item scores and is subtracted from 25 for even-numbered questions. In addition, the answers to the open-ended question were consistent with the SUS outcomes.

#### Qualitative Analysis

The thematic analysis data were organized hierarchically using a code frame to develop sentiment analysis by identifying patterns and themes. Based on the feedback received, 2 main themes emerged: positive and negative. The positive feedback theme comprised 3 subthemes: ease of use, clear language, and good audiovisual features. The negative feedback theme consisted of 4 subthemes: answering mandatory questions, lack of interaction, repetitive content, and below-average app features.

Positive and negative feedback are listed in Textbox 1.

Textbox 1. **Positive and negative feedback.**

**Positive Feedback (A)**

- Theme (A1) easy to use (number of mentions=7): feedback highlighted the user-friendly nature of the app, with participants (n=7) expressing appreciation for its simplicity and ease of use.
  - “The app was clear and easy to use.” [P1]
  - “It is my first time using such these kinds of applications. Once I understood the idea, then the uses were easy for me.” [P10]

- Theme (A2) clear language: (number of mentions=2): this theme reflected the clarity of the Arabic language and its integration into the app. [P3]
  - “Easy to use and the language is understandable and direct.”

- Theme (A3) good audiovisual features: number of mentions (n=2): positive feedback was received for the SelfBack app’s useful audio and visual features (n=2).
  - “The exercise pictures, sound, and instructions are clear.” [P7]

**Negative Feedback (B)**

- Theme (B1) answering the mandatory questions (number of mentions, n=4): participants (n=4) expressed their opinions on the mandatory and complicated process of having to ask consistent questions every time they use the app. They provided different feedback, including that it consumed too much of their time and led to frustration or quitting using the app altogether.
  - “Everything in the app is easy, but every time I log in, I have to answer the same question over and over.” [P3]

- Theme (B2) lack of interaction (number of mentions n=3): this theme pertained to the uncertainty around whether they are using the app correctly or whether they should stop using it. In addition, this feedback suggested that there was a lack of real interaction with health care practitioners (n=3).
  - “No interaction.” [P6]
  - “It is my first time using such these kinds of applications. Once I understood the idea, then the uses were easy for me. But I want someone to follow up with me.” [P10].

- Theme (B4) repetitive content (number of mentions, n=2): this theme concerned negative feedback regarding repeating instructions and exercises over time, regardless of LBP severity (n=2).
  - “It was beneficial, my comment is for how long should I have to use it? Because the app keeps repeating the same exercise.” [P9]

- Theme (B5) below expectations (number of mentions, n=1): this subtheme pertained to a user’s expectations of the app in comparison to other apps (n=1).
  - “The app was below my expectations and there is nothing special about it. There is another app with superior features that gives you more exercise and interaction.” [P11]

## Discussion

### Primary Findings

This study successfully translated and culturally adapted the English SelfBack app for the Saudi Arabian context, subsequently evaluating user satisfaction through a mixed-methods approach. The translation and adaptation process was efficiently executed, with an expert panel review identifying minimal discrepancies and confirming the absence of significant misunderstandings attributable to language. Usability testing with 11 participants yielded a positive overall usability rating. Qualitative data derived from semistructured interviews revealed a range of user perceptions. Positive feedback themes centered on the app’s intuitive interface, ease of navigation, clarity of the Arabic language implementation, and effective integration of audiovisual components. Conversely, negative feedback addressed concerns regarding the length of mandatory questionnaires, limited opportunities for user interaction, perceived redundancy in content delivery, and unmet expectations concerning the breadth and functionality of available features.

The usability and acceptability findings of this study resonate with prior SelfBack app assessments conducted in Aberdeen, Scotland and Trondheim, Norway [20], which also reported user feedback concerning desired improvements in app functionality and mixed reactions to motivational notifications. While the majority of Scottish participants appreciated the notifications they received, user reception in Norway was less favorable, with only half finding them useful. This variability in user response across different cultural contexts underscores the need for further refinement of the notification system to ensure its resonance with a broader range of users. This suggests that cultural adaptation of digital health interventions extends beyond language translation and requires careful consideration of user preferences and contextual factors related to how and when information is delivered. Future iterations of the notification system should consider incorporating user customization options, allowing individuals to select notification frequency, content, and delivery methods.

The requirement for mandatory sign-up questions during initial app interaction, while designed to collect data for personalized treatment plan updates, elicited negative feedback from some participants in this study, potentially contributing to user burden. Given that initial onboarding experiences can significantly influence app adherence [21], this step warrants streamlining and optimization to prioritize user comfort and engagement. This finding aligns with the broader literature on user experience in digital health, which emphasizes the importance of minimizing barriers to entry and maximizing user-friendliness, particularly during initial interactions. Future versions of the Arabic SelfBack app could explore simplifying the sign-up process, perhaps by reducing the number of mandatory fields or by clearly communicating the rationale behind the information requested to manage user expectations and prevent negative first impressions. Alternatively, a staged approach to data collection could be implemented, gathering essential information initially and allowing users to provide additional details later.

This study’s findings regarding the desire for personal interaction with health care professionals are consistent with a qualitative study by Alzahrani et al (2022) [21], which explored Saudi patients’ perspectives on mobile health apps. Their research revealed a preference for personal interaction with health care providers to enhance adherence and suggested incorporating social media groups for peer support within apps. This convergence of findings across studies highlights a potentially crucial cultural factor in the adoption and effective use of digital health interventions in Saudi Arabia. The desire for human connection and support, even within a digital context, may reflect cultural values emphasizing interpersonal relationships and community. These insights can inform future app development and design, suggesting that incorporating features that facilitate interaction with health care practitioners, such as secure messaging or telehealth integration, and fostering peer support communities within the app could significantly enhance its acceptability and effectiveness. Furthermore, addressing concerns regarding repetitive content and expanding the variety of exercises offered could further improve user engagement and satisfaction, aligning the app’s functionality more closely with user expectations and potentially leading to better outcomes.

While this study used a rigorous and evidence-based methodology for the translation and cultural adaptation of the SelfBack app, including a comprehensive expert review process, it is important to acknowledge certain limitations. The primary limitation lies in the relatively small sample size (N=11) used in the field usability testing. This limited sample size may restrict the generalizability of the findings and may not fully capture the diversity of experiences and perspectives within the target population. While the qualitative data gathered through interviews provided valuable insights into user perceptions, a larger and more representative sample would have strengthened the study’s conclusions regarding usability and acceptability. Furthermore, this study focused primarily on usability and user satisfaction; it did not assess the clinical effectiveness of the Arabic SelfBack app in managing LBP. Therefore, future research using a larger sample size and incorporating a robust evaluation of clinical outcomes, such as pain intensity, functional capacity, and quality of life, is warranted to determine the app’s efficacy in the Saudi Arabian context. Such research would provide a more comprehensive understanding of the app’s potential impact on LBP management and inform its wider implementation.

### Conclusions

This study successfully translated, culturally adapted, and evaluated the usability of the SelfBack app for Arabic-speaking users in Saudi Arabia, addressing a critical need for culturally sensitive digital health resources. Positive usability findings suggest the app’s potential for effective adoption and use. These results contribute to the growing evidence supporting mobile health for chronic pain management, particularly in underserved populations, and highlight the importance of cultural adaptation in digital health intervention development. The Arabic SelfBack app has the potential to improve access to evidence-based LBP self-management in Saudi Arabia. Future research should evaluate its clinical effectiveness to inform wider implementation and contribute to improved LBP care. This study also serves as a model for developing and adapting digital health interventions for other conditions and cultural contexts.

## Abbreviations

A-SUS: Arabic System Usability Scale
LBP: low back pain
NICE: National Institute for Health and Care Excellence
SUS: System Usability Scale
